# A cascade of classifiers for extracting medication information from discharge summaries

**DOI:** 10.1186/2041-1480-2-S3-S2

**Published:** 2011-07-14

**Authors:** Scott Russell Halgrim, Fei Xia, Imre Solti, Eithon Cadag, Özlem Uzuner

**Affiliations:** 1 University of Washington, PO Box 543450, Seattle, WA 98195, USA; 2 Cincinnati Children's Hospital Medical Center, Cincinnati, OH 45229-3039, USA; 3 University of Albany, SUNY, 135 Western Ave, Albany, NY 12222, USA

## Abstract

**Background:**

Extracting medication information from clinical records has many potential applications, and recently published research, systems, and competitions reflect an interest therein. Much of the early extraction work involved rules and lexicons, but more recently machine learning has been applied to the task.

**Methods:**

We present a hybrid system consisting of two parts. The first part, field detection, uses a cascade of statistical classifiers to identify medication-related named entities. The second part uses simple heuristics to link those entities into medication events.

**Results:**

The system achieved performance that is comparable to other approaches to the same task. This performance is further improved by adding features that reference external medication name lists.

**Conclusions:**

This study demonstrates that our hybrid approach outperforms purely statistical or rule-based systems. The study also shows that a cascade of classifiers works better than a single classifier in extracting medication information. The system is available as is upon request from the first author.

## Background

Narrative clinical records store patient medical information, and extracting this information is an important problem with practical application [1]. In this work we describe a system for extracting detailed medication information from hospital discharge summaries using a combination of rules and statistical learning.

### Related work

Until recently, much of the work done on extracting medication information from clinical documents involved rules and lexicons. Gold et al. used a set of parsing rules formatted as regular expressions and a drug name lexicon [2], while Xu et al. filled a semantic representation model using lexicon lookups, regular expressions, and disambiguation rules [3]. While convenient in the absence of a large corpus of annotated data, such rule-based systems can be time-consuming to build and difficult to manage [4]. More recently, machine learning has been applied to the task: Patrick and Li used a conditional random fields (CRF) named entity identifier and a support vector machine (SVM) relationship classifier [5], Tikk and Solt also employed CRF to finding named entities [6], and Li et al. worked with AdaBoost and CRF [7].

Maximum Entropy (MaxEnt) is a machine learning algorithm that, in the biomedical domain, has been used to identify personally identifiable information [8] and assign gene function codes to genes [9]. In information extraction, Chieu and Ng used it to extract succession management templates [10]. As far as we know, MaxEnt has not been applied to medication information extraction in the clinical domain.

### The problem task

For this work we are interested in the automatic extraction of information about medications that a patient takes. Specifically, we extract the following fields from hospital discharge summaries: names of medications (m), dosages (do), modes (mo), frequencies (f), durations (du), and reasons (r) for taking these medications. We refer to the medication field as the name field and the other five fields as the non-name fields. All non-name fields should be linked to exactly one name field in the system output. A name field and all the non-name fields that link to it form one or more *entries*, each of which corresponds to a medication event. An entry appears in either a list of medications (“list”) or in narrative text (“narrative”). Table 1 shows an excerpt from a discharge summary and the corresponding entries in the gold standard. The first entry appears in narrative text, and the second in a medication list.

**Table 1:** A sample discharge summary excerpt and the corresponding entries in the gold standard

**Excerpt of Discharge Summary** 55 the patient noted that he had a recurrence of this 56 vague chest discomfort as he was sitting and 57 talking to friends. He took a sublingual 58 Nitroglycerin without relief. ... 65 Flomax ( Tamsulosin ) 0.4 mg, po, qd,...
**Gold standard**: m=“Nitroglycerin” 58:0 58:0 ||do=“nm”|| mo=“sublingual” 57:6 57:6 ||f=“nm” ||du=“nm” || r=“vague chest discomfort” 56:0 56:2 || ln=”narrative” ... m="flomax ( tamsulosin )" 65:0 65:3||do="0.4 mg" 65:4 65:5||mo="po" 65:6 65:6||f="qd" 65:7 65:7||du="nm"||r="nm"||ln="list"

The fields inside an entry are separated by “||”. Each field is represented by the string and its position (i.e., “line number: token number” offsets). “nm” means the field value is not present for this medication event. The fields are medication name (m), dosage (do), mode (mo), frequency (f), duration (d), and reason (r).

In this paper we present our approach to this task. A pre-processor generates section information via regular expressions and part-of-speech tags from the Stanford tagger [11]. The next step is the system’s core: a cascade of statistical classifiers that identify medication fields. Simple rules then form entries from these fields.

### Data sets

The data for training and evaluating our methods came from the 2009 i2b2 challenge [12]. The challenge organizers released 696 summaries for system development; a gold standard for entries was provided for 17 of them. The University of Sydney team [5] annotated 145 of the 696 summaries and generously shared their annotations with i2b2 after the challenge for future research. We obtained and used 110 of those annotations as our training set and the remaining 35 as our development set. After the challenge, 251 more summaries were annotated by the challenge participants, and those summaries formed the final test set on which our system was evaluated.

The sizes of the data sets used in our experiments are shown in Table 2. The average number of entries and fields vary across the sets because the summaries in the test set were chosen randomly from a set of 547 held-out summaries, whereas the University of Sydney team chose to annotate the longest summaries in the released set.

**Table 2:** The data sets used in our experiments

Data Sets	# of Summaries	# of Entries	# of Fields	# of Name	# of Dose	# of Freq	# of Mode	# of Duration	# of Reason
Training set	110	5970 (54.3)	14886 (135.3)	5684 (51.7)	2929 (26.6)	2740 (24.9)	2146 (19.5)	302 (2.7)	1085 (9.9)
Dev set	35	2401 (68.6)	5988 (171.1)	2302 (65.8)	1163 (33.2)	1096 (31.3)	880 (25.1)	111 (3.2)	436 (12.5)
Test set	251	8936 (35.6)	22041 (87.8)	8495 (33.8)	4387 (17.5)	3999 (15.9)	3307 (13.2)	511 (2.0)	1342 (5.3)

The numbers in parentheses are the average numbers of entries or fields per discharge summary.

## Methods

We developed a hybrid system with three processing steps: (1) a pre-processing step, (2) a field detection step that identifies the six fields, and (3) a field linking step that links fields together to form entries. The second step is a statistical system, whereas the other two steps are rule-based. The second step was the main focus of this study. The entire system was first presented at the 2010 Louhi Workshop [13], where the authors were invited for the special issue of this journal.

### Pre-processing

In addition to common processing steps such as part-of-speech (POS) tagging, our pre-processor includes a section segmenter that breaks discharge summaries into sections. Discharge summaries tend to consist of sections such as “ADMIT DIAGNOSIS”, “PAST MEDICAL HISTORY”, and “DISCHARGE MEDICATIONS”. Knowing section boundaries is important for the task because, according to the i2b2 challenge annotation guidelines for creating the gold standard, medications occurring under certain sections (e.g., “FAMILY HISTORY” and “ALLERGIES”) were to be excluded from the system output. Knowing the sections could also be useful for field detection and linking. For example, the ‘DISCHARGE MEDICATIONS’ section is more likely to contain medications in a list than medications embedded in narrative text.

The set of sections and the exact spelling of section headings vary across discharge summaries. The section segmenter uses a regular expression (a line starting with a sequence of capitalized letters followed by a colon) to collect potential section headings from the training data. The headings whose frequencies are higher than a threshold are used to identify section boundaries in the discharge summaries.

### Field detection

This step consists of three modules: *find_name*, which finds medication names, *context_type*, which determines whether each identified medication name appears in narrative text or in a list of medications, and *find_others*, which detects the five non-name field types. For all three modules we use the Maximum Entropy (MaxEnt) learner in the MALLET package [14] because the training time for MaxEnt can be shorter than more sophisticated algorithms such as CRF [15]. For *find_name* and *find_others*, we follow the common practice of treating named entity (NE) detection as a sequence labeling task with the Inside-Outside-Beginning (IOB) tagging scheme; that is, each token in the input is tagged with B-x (beginning an NE of type x), I-x (inside an NE of type x) and O (outside any NE).

### The *find_name* module

As this module identifies medication names only, the tagset under the IOB scheme has three tags: B-m for beginning of a name, I-m for inside a name, and O for outside.

Various features are used for this module, which we group into four types:

• (F1) includes word n-gram features (n=1,2,3). For instance, the bigram w_i-1_ w_i_ looks at the bigram consisting of the previous word and the current word.

• (F2) contains features of properties of the current word and its neighbors (e.g., their POS tags, affixes, lengths, containing section, capitalization, etc.)

• (F3) checks the IOB tags of previous words

• (F4) contains features that check whether an n-gram in the text appears as part of a medication name in some medication name lists.

For (F4) we used two medication name lists. The first list consists of medication names from the training data and is the only list used in set F4a. The second list includes drug names from the FDA National Drug Code Directory (http://www.accessdata.fda.gov/scripts/cder/ndc/) and is used to test whether features that check an external resource improve performance. Feature set F4b uses both lists.

### The *context_type* module

This module is a binary classifier that determines whether a medication name occurs in a list or narrative context. Features used by this module include the section name as identified by the pre-processing step, the number of commas and words on the line, the medication name itself and its position on the line, and nearby words.

### The *find_others* module

This module complements the *find_name* module and uses eleven IOB tags to identify five non-name fields. The feature set used in this module is similar to the one used in *find_name*, but some features in (F2) and (F4) are modified to suit the non-name fields. For instance, one feature that was not present in *find_name* checks whether a word fits a common pattern for dosage. In addition, some features in *find_others* look at the output of previous modules, like the location of nearby medication names, as this information can be provided by the *find_name* module at test time.

### Field linking

The final step is to form entries by associating each medication name with its related fields. Our current implementation uses simple heuristics. First, for each non-name field the closest prior and subsequent name fields are identified. Second, each non-name field is linked to one of those two name fields. In most cases, the non-name field is linked to the prior name field, but if the distance to the subsequent name field is shorter than the distance to the prior name field by more than two lines, we link the non-name field to the subsequent name field. Third, the (name, non-name) pairs are assembled into entries with a few rules that apply if more than one non-name field of the same type is linked to the same name field. More information about the modules, including the features and the linking rules, is available in [16].

## Results

In this section, we report our system’s performance on the development and test sets.

### Evaluation metrics

We use two sets of evaluation metrics: horizontal and vertical. Horizontal metrics measure performance at the entry level, whereas vertical metrics measure performance at the field level. Both metrics compare fields between the system output and the gold standard for an exact match. A field in the system output *exactly matches* a field in the gold standard if the two fields’ spans are identical and they have the same field type [12]. The primary metric for the i2b2 challenge was horizontal F-score, which is the metric we use in this section unless otherwise specified.

### Statistical significance

To determine whether the difference between two systems’ performances is statistically significant, we use approximate randomization tests [17]. Given two systems that we would like to compare, we first calculate the difference between horizontal F-scores. Then two pseudo-system outputs are generated by swapping (at 0.5 probability) the two system outputs for each discharge summary. These new pseudo-sets are scored as normal, and the difference between F-scores calculated. If the difference between F-scores of these pseudo-outputs is no less than the original F-score difference, a counter, *i*, is increased by one. This process is repeated *n*=10,000 times, and the p-value of the significance is equal to (*i*+1)/(*n*+1). If the p-value is smaller than a predefined threshold (e.g., 0.05), we conclude that the difference between the two systems is statistically significant. A conservative statistical correction (Bonferroni) was used to adjust for multiple significance comparisons.

### Performance of the field detection step

Table 3 shows the vertical precision, recall, and F-score on identifying the six field types in the development set, using all 110 training files and the F1-F4b feature sets. Table 3 shows that, while the system detects most fields well, it has trouble with “duration” and “reason,” and particularly with the recall of those fields.

**Table 3:** The performance of field detection on the development set

	Precision	Recall	F-score
Name	91.2	88.5	89.9
Dosage	96.6	90.8	93.6
Frequency	93.9	89.0	91.8
Mode	95.7	90.3	92.9
Duration	73.8	43.2	54.5
Reason	72.2	31.0	43.3
All fields	92.6	84.5	88.4

Vertical precision, recall, and F-score.

When making the “narrative” vs. “list” distinction, the accuracy of *context_type* is 95.4%. In contrast, the accuracy of the baseline (which assigns a “list” context to each medication name) is only 55.6%.

### Performance of the field linking step

In order to evaluate the field linking step, we generated a list of unique (name, non-name) pairs from the gold standard where the name and non-name fields appear in the same entry. We then compared the fields in these pairs with the ones produced by the field linking step for exact matches and calculated precision, recall, and F-score. Table 4 shows the results of two experiments: in the gold standard input experiment, the input to the field linking step is the fields from the gold standard, which allows us to evaluate the linker directly assuming the field detection step is perfect; in the system input experiment, the input is the actual output of our system’s field detection step. Both experiments were performed on the development set. This table shows that our heuristics perform well when given perfect input, but perform considerably worse when given the imperfect fields as detected by the system as input.

**Table 4:** The performance of the field linking step on the development set

Input	Precision	Recall	F-score
Gold standard	87.4	75.1	80.8
System	96.2	94.5	95.3

Gold standard input: assuming perfect input from the field detection step; System input: using the actual output of the system’s field detection step.

### Effect of feature sets

To test the effect of feature sets on system performance, we trained the *find_name* and *find_others* modules with different feature sets. The models were trained on the training set and the system was tested on the development set.

The results are in Table 5. For the last two rows, the F1-F4a row uses a medication name list derived from the training data and the F1-F4b row adds the FDA’s National Drug Code Directory list. The F-score difference between all adjacent rows is statistically significant at *p*≤0.05, except for the pair F1-F3 vs. F1-F4a. It is not surprising that using the first medication name list on top of F1-F3 does not improve the performance, as the same kind of information has already been captured by F1. The improvement of F1-F4b over F1-F4a shows that the system can incorporate additional resources and achieve a statistically significant gain.

**Table 5:** System performance on the development set with different feature sets

Features	Precision	Recall	F-score
F1	72.5	60.3	65.8
F1-F2	82.5	78.2	80.3
F1-F3	88.4	77.9	82.8
F1-F4a	87.4	77.9	82.4
F1-F4b	88.1	79.4	83.5

Horizontal precision, recall, and F-score demonstrates that including all proposed feature sets and the external data lists results in the best performance.

### Results on the test data

Table 6 shows the system performance on the test data. This includes the horizontal precision, recall, and F-score, as well as the vertical metrics. The system was trained on the union of the training and development data. These results are good overall, and confirm our findings on the development set that the system has difficulty finding “duration” and “reason” fields. Despite the poor performance on these fields, the vertical “all fields” scores are still closer to those of the other four fields, reflecting the sparseness of the challenging fields in the data.

**Table 6:** System performance on the test set

	Field	Precision	Recall	F-score
Horizontal	N/A	88.6	80.2	84.1
**Vertical**	Name	92.6	87.1	89.8
	Dosage	96.3	90.2	93.1
	Frequency	95.6	90.8	93.2
	Mode	96.7	90.2	93.3
	Duration	70.6	40.5	51.5
	Reason	73.4	34.7	47.1
	
	All fields	91.6	82.7	86.9

Horizontal and vertical metrics for system trained on the union of the training and development sets using all features in sets F1-F4b.

## Discussion

### Field detection

As mentioned, the results for “duration” and “reason” are the lowest of all fields, which was also the case for all the participating systems in the challenge [12]. Those two fields are also the most difficult for humans to annotate, as indicated by their low inter-annotator agreement [18]. One possible reason for these fields’ difficulty is that their content varies considerably more than that of “mode” and “frequency” [12]. Another possibility is that, because they are longer and have more variability in their length than other fields, it is more difficult to locate their exact boundaries [16].

### Field linking

The results shown in Table 4 are intriguing. The linking rules appear to be adequate when given perfect input, but perform worse when operating on the imperfect input from the system’s field detection module. It is unclear how much of the drop in performance is due to the rules themselves and how much is due to the limiting factor of the imperfect fields. One way to explore this in future work would be a manual effort to construct the best possible set of entries given the system-defined fields and evaluate those entries against the gold standard.

### Effect of training data size

**Figure 1:**
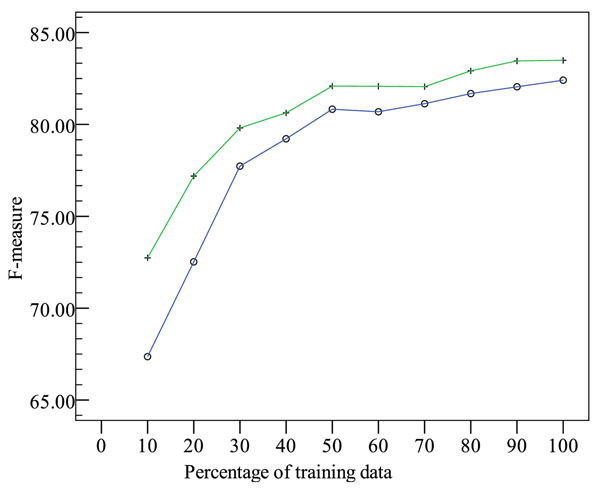
**System performance on the development set with different training set sizes.** Key: + represents horizontal F-scores with features in F1-F4b; ○ represents horizontal F-scores with features in F1-F4a.

Figure 1 shows the system performance on the development set when different portions of the training set are used for training. The curve with “+” signs represents the results for F1-F4b, and the curve with circles represents the results for F1-F4a.

The figure illustrates that, as the training data size increases, the horizontal F-score with both feature sets improves. In addition, the external list is most helpful when the training data size is small, as indicated by the decreasing gap between the two curves.

### Cascade vs. f*ind_all*

Using three separate modules for field detection allows each one to use the features most appropriate for it. In addition, later modules can use features based on the output of previous modules. However, a potential downside is errors propagating through the cascade. An alternative is to use a single module to detect all six field types.

We built and tested such an alternative, which we call *find_all*. This module eliminates *find_name* and *context_type*. It finds medication names by adding two more class labels to *find_others*: B-m and I-m. Thus it is a 13-way MaxEnt classifier that can find all six field types in one pass through the text.

**Figure 2:**
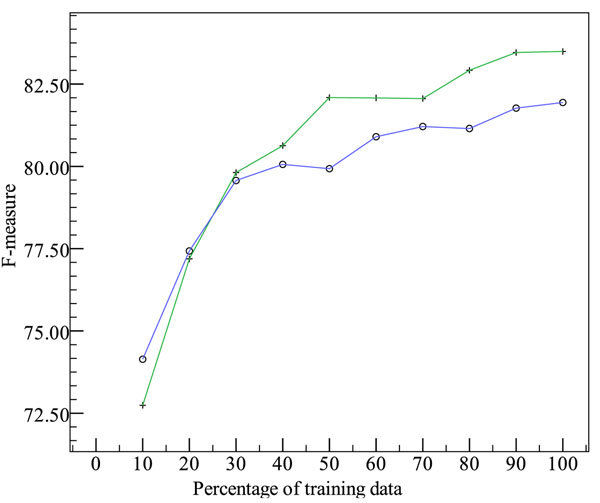
**Cascade vs. *find_all* for field detection on the development set.** Key: + represents horizontal F-scores with the three-module cascade; ○ represents horizontal F-scores with *find_all*.

Figure 2 compares the horizontal F-score of the system using the *find_all* algorithm as its field detection step with that of the system with cascading modules. Both use the F1-F4b feature sets except that, since *find_others* uses some features that check the output of previous modules which are not available to *find_all*, such as the look-ahead proximity of name fields, those features have been removed from *find_all*. Both algorithms are trained on the training set and evaluated on the development set.

Interestingly, when 10% of the training set is used for training, *find_all* has a higher F-score than the cascading approach, although the difference is not statistically significant at *p*≤0.05. As more data is used for training, the cascade outperforms *find_all*, and the difference between the two is statistically significant at *p*≤0.05 when at least 50% of the training data is used. One possible explanation for this phenomenon is that as more training data becomes available, the early modules in the cascade make fewer errors; as a result, the disadvantage of potential error propagation in the cascading approach is outweighed by the advantage that the later modules can use features that check the output of the earlier modules.

### I2b2 challenge entrants as benchmark

Strictly for purposes of providing a benchmark, we report the horizontal precision, recall, and F-score on the test set of the top five systems [5][19][20][21][6] that participated in the 2009 i2b2 challenge [12] in Table 7. The table shows that the performance of our system is comparable to the top systems in the i2b2 challenge.

**Table 7:** Benchmark performances of the top five i2b2 systems on the test set

Rank	Team	Precision	Recall	F-score
1	USyd	89.6	82.0	85.7
2	Vanderbilt	84.0	80.3	82.1
3	Manchester	86.4	76.6	81.2
4	NLM	78.4	82.3	80.3
5	BME-Humboldt	84.1	75.8	79.7

Horizontal precision, recall, and F-score.

A caveat of comparing Tables 6 and 7 is that time, availability of training data, and differences in available resources make it difficult to compare these systems to one another. First, as non-entrants in the challenge, we had more time to work on our system than the other systems cited here. Mork et al. report that their entry into the challenge used simple rules and lookup-lists due to time constraints [21]. Second, there was a disparity in the amount of data used. While teams were allowed to annotate their own training set, only one team in the top five did: the University of Sydney team [5]. This disparity in data may also explain why, of the top five performing systems, only one used any kind of machine learning. As the University of Sydney graciously shared their data, we were able to emphasize machine learning in our approach. In fact, both the Spasić et al. [20] and Tikk and Solt [6] teams reported that they implemented a rule-based system with lexicons because of the small amount of training data provided. Finally, teams were allowed to use any resource, including existing systems and lexicons unavailable to the general public. Doan et al. applied their existing rule-based medication extraction system to the problem and placed second in the challenge [19]. These variations in resources made the challenge similar to the so-called open-track challenge in the general NLP field and complicate head-to-head comparisons.

## Conclusions

We present a hybrid system for medication information extraction. It is built around a series of cascading MaxEnt classifiers for field detection. Its performance compares favorably to systems approaching the same task with rules and other machine learning algorithms. Incorporating additional resources as features improves performance. Given enough training data, the cascade system outperforms a single classifier that finds all fields at once. In the future, we plan to try to improve scores on the “duration” and “reason” fields by adding more specialized classifiers. We also plan to replace the rule-based linking module with a statistical linker to improve results.

## Competing interests

Authors IS and OU were part of the i2b2 challenge organizing committee.

## Authors' contributions

SRH designed and implemented the system and, with FX, designed the experiments and drafted the paper. IS, EC, and OU prepared the data sets and, with FX, designed the i2b2 challenge. EC did the statistical significance testing. All authors contributed to the final manuscript.
